# Voltamperometric Sensors and Biosensors Based on Carbon Nanomaterials Used for Detecting Caffeic Acid—A Review

**DOI:** 10.3390/ijms21239275

**Published:** 2020-12-04

**Authors:** Alexandra Virginia Bounegru, Constantin Apetrei

**Affiliations:** Department of Chemistry, Physics and Environment, Faculty of Sciences and Environment, “Dunărea de Jos” University of Galaţi, 47 Domnească Street, 800008 Galaţi, Romania; alexandra.meresescu@ugal.ro

**Keywords:** caffeic acid, voltamperometric sensor, biosensor, carbon nanomaterials

## Abstract

Caffeic acid is one of the most important hydroxycinnamic acids found in various foods and plant products. It has multiple beneficial effects in the human body such as antioxidant, antibacterial, anti-inflammatory, and antineoplastic. Since overdoses of caffeic acid may have negative effects, the quality and quantity of this acid in foods, pharmaceuticals, food supplements, etc., needs to be accurately determined. The present paper analyzes the most representative scientific papers published mostly in the last 10 years which describe the development and characterization of voltamperometric sensors or biosensors based on carbon nanomaterials and/or enzyme commonly used for detecting caffeic acid and a series of methods which may improve the performance characteristics of such sensors.

## 1. Introduction

Caffeic acid (CA) (3,4-dihydroxycinnamic acid) is one of the main phenolic derivatives, usually found in red wine, green tea, coffee, fruits, and vegetables [1,2,3]. An exceptional source of free or bound CA is the fruit of the plant called wild cherry (chokecherry), *Prunus virginiana*, native to North America [4]. CA plays an extremely important role in human life, having antibacterial and anti-inflammatory effects [5,6]. However, excessive intake of CA can cause negative side effects [6]. Therefore, it is important to quantitatively determine the concentration of CA in foods, dietary supplements, and pharmaceuticals. In recent years, a wide variety of electrochemical sensors and biosensors having different performance characteristics and implying specific manufacturing processes [7] have been developed for the quantitative analysis of CA [8,9,10]. Raising awareness regarding the state of research in this field is important and it is the objective of this review.

## 2. Caffeic Acid and Its Role in the Human Body

CA is a phenolic compound resulting from the secondary metabolism of plant products [11,12,13] such as olives, coffee beans, fruits, potatoes, carrots, and propolis and it is the main hydroxycinnamic acid used in human diets [14,15,16,17]. Caffeic acid is found in the form of a monomer, as esters with organic acids, amides, and glycosides [18] or in more complex forms, such as dimers, trimers, and flavonoid derivatives. It may also be bound to proteins and other polymers in the cell wall of plant products [19].

**Figure 1:**
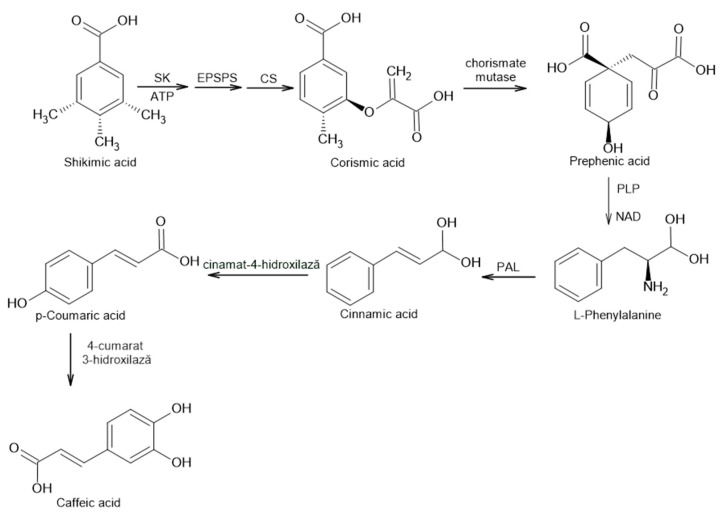
The biosynthesis of CA (figure made by using ChemDraw—http://www.perkinelmer.com/category/chemdraw) [20].

CA, 3-(3,4-dihydroxyphenyl)-2-propenoic acid, has a phenylpropanoid structure (C6-C3) with a 3,4-dihydroxylated aromatic ring attached to a carboxylic acid by the *trans*-ethylene bond [20,21]. The biosynthesis of this compound in plants takes place on the endogenous shichymate pathway which is responsible for the production of aromatic amino acids [22,23]. The reaction begins with the chemical acid and undergoes three enzymatic reactions: the first is a phosphorylation mediated by the enzyme schymatokinase, followed by the conjugation of a molecule of phosphenolpyruvate, mediated by the enzyme 5-enolpyruvylchychimate-3-phosphate (EPSP) and, finally, by the enzyme chorismatase, which reaches one of the most important intermediate metabolites of this pathway, i.e., chorismic acid [20,21,22,23,24]. It is converted to prefenic acid from chorismic acid (a precursor of L-phenylalanine) under the action of chorismate mutase. L-phenylalanine formation is mediated by pyridoxal 5-phosphate (PLP) as a coenzyme in the deamination process and nicotinamide adenine dinucleotide (NAD) responsible for electron transfer [20,24]. Deamination of L-phenylalanine by phenylalanine ammonium lyase (PAL) leads to the formation of cinnamic acid which is converted to p-coumaric acid using the enzyme cinnamate-4-hydroxylase (C4H) and finally to CA by the enzyme 4-coumarin 3-hydroxylase (C3H) [20]. The biosynthesis of the CA is presented in Figure 1.

**Figure 2:**
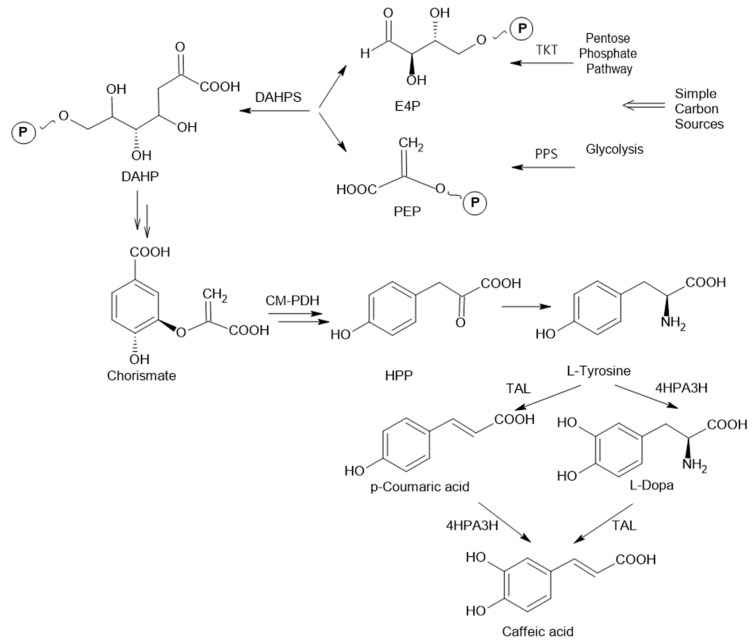
Proposed caffeic acid (CA) biosynthetic pathway. Native tyrosine biosynthetic pathway in *E. coli* and the artificial dual pathway mediated by 4HPA3H and TAL for CA biosynthesis from tyrosine. PPS: phosphoenolpyruvate synthase; TKT: transketolase; CM-PDH: chorismate mutase-prephenate dehydrogenase; DAHPS: 3-deoxy-D-arabino-heptulosonate-7-phosphate synthase; 4HPA3H: 4-hydroxyphenylacetate 3-hydroxylase; TAL: tyrosine ammonia lyase; E4P: D-erythrose-4-phosphate; PEP: phosphoenolpyruvate; HPP: 4-hydroxyphenylpyruvate [23].

This compound may be obtained in large quantities by organic synthesis [25]. Genetic changes in microorganisms, such as *Escherichia coli* strains [26,27] have led to the production of two enzymes: 3-hydroxylase hydroxyphenylacetate (4HPA3H) and tyrosine ammonium lyase (TAL) which act on the production of L-tyrosine, a p-coumaric acid and L-dopa, respectively. A new action of these enzymes on the two intermediate molecules leads to the production of CA [20]. The proposed CA biosynthetic pathway is presented in Figure 2.

CA participates in the defense mechanism of plants against predators, pests, and infections, because it has an inhibitory effect on the growth of fungi and bacteria [28,29]. In vitro and in vivo experiments have demonstrated that CA and its derivatives have numerous beneficial physiological effects, such as antibacterial [30,31,32], antiviral [33,34,35,36], antioxidant [37,38,39,40], anti-inflammatory [5,41,42,43], anti-atherosclerotic [44,45,46], immunostimulatory [47,48,49], antidiabetic [50,51], cardioprotective [52,53], antiproliferative [54], hepatoprotective [55,56], anticancer [20,54,57,58,59], etc.

CA plays an important role in the human body due to its numerous beneficial effects mentioned above. It may also be found in pharmaceuticals [60,61,62]. However, there are studies which show that high doses of CA may cause significant side effects on living things, for example preventing the implantation of embryos [63] or even cancerous effects [64]. The fact is known that a large proportion of CA is absorbed in the small intestine and enters the bloodstream in a large proportion [65]. Some research has shown that CA may favor the development of squamous cell tumors in the stomach and kidneys of rats and mice [64]. In 2019, researchers studied the toxicity of CA for the reproductive function and later in the development of offspring in female mice. In the three-segment study, female mice were continuously exposed to 0, 0.15, 5, or 150 mg/kg/day of CA by gavage. Doses of 5 and 150 mg/kg/day of CA were found to affect embryo implantation when administered before the sixth day of gestation. In addition, 150 mg/kg/day of CA affected fetal weight gain [63].

As there is a risk of overdose and/or of side effects, research has focused on new, accurate methods for determining CA. Up to the present, various analytical methods have been developed for CA determination which include liquid chromatography, capillary electrophoresis, as well as electrochemical methods [7,66,67,68,69]. Among the methods mentioned, the electrochemical ones are preferred due to their excellent sensitivity, fast response, good selectivity, low cost, reliability, and simplicity [1,69]. In the case of electrochemical techniques, the selective determination depends mainly on the electroactivity of the analyte, but the sensitive material also influences this type of determination. Many materials used for CA detection have been studied and improved, such as carbon nanomaterials, metal oxides/hydroxides, metal nanoparticles, and conductive polymers [70,71,72,73,74].

## 3. Sensors and Biosensors Used in the Detection of Caffeic Acid

Electrochemical sensors and biosensors are portable and sensitive devices used for the rapid and cost-effective determination of analytes ranging from metal ions to organic compounds, pollutants, proteins, antigens, deoxyribonucleic acids (DNAs), viruses, bacteria, and others [75,76,77,78,79]. Electrochemical biosensors are analytical devices similar to electrochemical sensors, but they incorporate biological molecules for rapid and accurate detection of target species [80,81]. Voltammetric methods may be used for both sensors and biosensors and are applicable to electroactive compounds, such as caffeic acid.

The main sensors based on carbon nanomaterials and biosensors based on carbon nanomaterials and enzymes which use voltammetric methods for detecting caffeic acid will be presented in the following sections of this paper.

### 3.1. Sensors Based on Carbon Nanotubes

Due to their excellent mechanical and electrical properties, carbon nanotubes have become one of the most popular carbon nanomaterials. Carbon nanotubes are cylindrical tubes made of carbon atoms with an inner diameter of >0.9 nm. Heat treatment is required to change the structure of tubes from a single-walled carbon nanotube (SWNT) to double-walled (DWNT) or even multiwalled (MWNT) carbon nanotubes. Both DWNT and MWNT are much more stable as compared to SWNT [82].

**Scheme 1:**
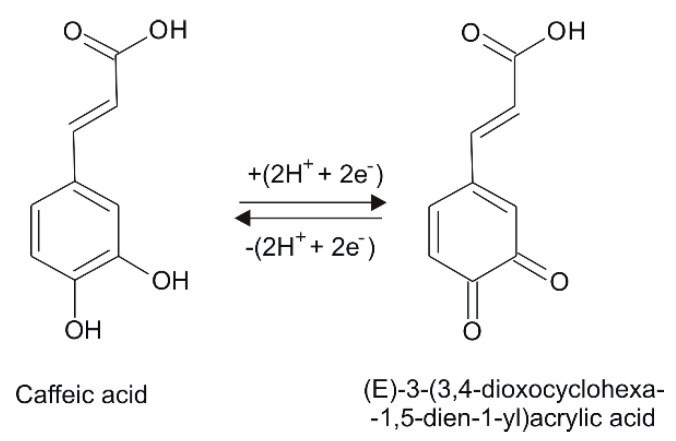
The reversible oxidoreduction process of caffeic acid at the sensitive element of an electrochemical sensor.

V. Erady et al. describe a voltammetric sensor based on carbon paste modified with multilayer carbon nanotubes, bismuth, and cetyltrimethylammonium bromide for the quantification of CA. Bismuth-modified multilayer carbon nanotubes obtained by using the technique of casting and cetyltrimethylammonium bromide ensure the improvement of the carbon paste electrode surface [8]. The detection technique used was differential pulse voltammetry (DPV). Several experimental parameters were studied such as pH and scan rate. Optimal results were obtained at physiological pH and the response was linear in the range 6.0 × 10^−8^–5.0 × 10^−4^ M. The limit of detection and quantification limits (0.157 and 1.910 nM (S/N = 3), respectively) proved to be good. Very good results were also obtained for the detection of CA in samples such as coconut water, tea, and fruit juices, without necessarily pretreating the sample. CA electrooxidation took place with the involvement of a low activation energy, the technology used for sensor preparation was light, and the materials used were safe in terms of toxicity [8]. The detection mechanism of the electrochemical sensors on the detection of caffeic acid is shown in Scheme 1.

It can be observed that the redox process is reversible and involves the exchange of two protons and two electrons. CA is electrochemically oxidized to the *o*-quinone derivative, (E)-3-(3,4-dioxocyclohexa-1,5-dien-1-yl)acrylic acid. The process is reversible, the *o*-quinone derivative is reduced to caffeic acid. This reversible process is well evidenced when the detection technique is the cyclic voltammetry [70].

Another paper makes reference to the use of nitrogen-doped carbon quantum dots (N-CQD) modified with a hexagonal structure composite of porous copper oxide (N-CQD/HP-Cu_2_O) and multilayer carbon nanotubes. The composite was synthesized by using a simple hydrothermal method. The N-CQD/HP-Cu_2_O composite was evaluated by using infrared spectroscopy, powder X-ray diffraction, electron scanning microscopy, and transmission electron microscopy. Next, the N-CQD/HP-Cu_2_O composite was modified with multilayer carbon nanotubes (MWCNT) by using the ultrasonication method, in order to obtain the hybrid composite N-CQD/HP-Cu_2_O/MWCNT. This composite is used as a sensitive material for electrode modification which electrochemically determines the CA. Cyclic voltammetry, differential pulse voltammetry, and electrochemical impedance spectroscopy were used as electrochemical techniques. The hybrid composite proved to be promising for electrocatalytic applications. Finally, the developed sensor was used to detect CA in red wine samples, and the results were satisfactory.

N-CQD/HP-Cu_2_O/MWCNT/GCE proved to be much more effective as compared to Cu_2_O/GCE, N-CQD/HP-Cu_2_O/GCE and unmodified GCE. Moreover, there was an increase in current and a shift of the oxidation potential to lower values as compared to the unmodified GCE. Due to the porous nature of the modified surface, to the synergistic effect of the hybrid material, to the higher stability, and to the improved electrocatalytic activity, CA detection was performed with a low detection limit (0.004 μM) and a high sensitivity (31.85 μA·μM^−1^·cm^−2^) [83].

Modifying sensors by using different materials leads to better electrochemical responses, with higher sensitivity and higher detection limit. In another paper, a vitreous carbon electrode modified with a titanium dioxide composite and carbon nanotubes (Ce-TiO_2_/CNTs) doped with cerium (Ce-TiO_2_/CNTs/GCE). Ce-TiO_2_/CNTs/GCE was used to determine CA. The electrochemical behavior of the sensor was studied, and the results indicated that CeTiO_2_/CNTs/GCE show a higher current intensity for CA oxidation, as compared to GCE, CeTiO_2_/GCE and CNTs/GCE. Combined with the good catalytic ability of Ce-TiO_2_ and the large specific surface area, as well as with the excellent electrical conductivity of CNTs, Ce-TiO_2_/CNTs demonstrate excellent electrochemical detection of CA. Under optimal conditions, the intensity of the oxidation peak of Ce-TiO_2_/CNTs/GCE had a linear increase directly proportional to the concentration of CA in the range of 1.0 nM–10.0 μM [84].

A new photoelectrochemical sensor (PEC) was developed for the determination of CA based on titanium dioxide (TiO_2_) nanoparticles, carbon nanotubes (CNTs), and cadmium telluride quantum dots (CdTeQDs). This PEC sensor showed a high photocurrent in the presence of CA under the incidence of visible light as compared to each individual material used in the construction of the composite material. Material characterization was performed by X-ray diffraction (XRD), scanning electron microscopy (SEM), linear scanning voltammetry (LSV), electrochemical impedance spectroscopy (EIS), and amperometry. Under optimized experimental and operational conditions, the photocurrent of the PEC sensor showed a linear relationship with the increase of the CA concentration from 0.5 to 360 μM and a detection limit of 0.15 µM. The PEC sensor has been successfully used for the determination of CA in tea and coffee samples. The recovery values for CA of 99.9% and 97.4%, respectively, in coffee and tea samples, suggested a good accuracy of the proposed method. In addition, the sensor has demonstrated excellent accuracy, selectivity, and reproducibility for CA determination [85].

S. Sakthinathan et al.’s work describes a 3D nano-composite of graphene and carbon nanotubes (3DG/MWCNTs-Nc) which was synthesized by a simple hydrothermal method for the amperometric determination of CA. The prepared nanocomposite was characterized by scanning electron microscopy (SEM), ultraviolet (UV) spectroscopy, Raman spectroscopy, and infrared (IR) spectroscopy. Furthermore, the electron transfer properties at the interface of the modified electrode were studied by electrochemical impedance spectroscopy (EIS). The electrochemical behavior of the modified electrode was studied by cyclic voltammetry (CV) and amperometric technique (i-t). The proposed electrode showed excellent electrocatalytic activity for CA detection. Under optimal conditions, the 3DG/MWCNTs-Nc electrode showed a linearity range of 0.2–174 μM, a detection limit (LOD) of 17.8 nM, and a sensitivity of 5.8308 μA·μM^−1^·cm^−2^. The applied potential was +0.2 V. Following the analysis of the voltammetric responses, the modified electrode 3DG/MWCNTs-Nc showed good repeatability, reproducibility, and stability. In addition, the manufactured electrode was successfully used to determine CA in real samples with satisfactory RSD. All the results suggest that 3DG/MWCNTs-Nc is suitable for electrochemical determination of CA [86].

In the last year, R. Nehru and collaborators have developed an extremely sensitive sensor by depositing on the surface of the vitreous carbon electrode the composite f-MWCNTs/a-NaFeO_2_, prepared by ultrasonication. The purpose of the modified sensor was the electrochemical determination of CA. The microstructural characteristics of the f-MWCNTs/-NaFeO_2_ composite were analyzed by different physico-chemical methods and analytical techniques. Cyclic voltammograms of the sensor modified with f-MWCNTs/-NaFeO_2_ composite showed electrochemical detection of CA. Sensitive performance was improved due to the modification of the electrode with the -NaFeO_2_-fMWCNT composite obtained by ultrasonication. Under optimal conditions, the developed sensor showed ultrasensitivity (44.685 μA·μM^−1^·cm^−2^), excellent linearity (R^2^ = 0.9943), and a low detection limit (LOD = 0.002·μM) using DPV. The developed sensor can improve the determination of CA in various antioxidant solutions, being a real support for the medical system [69].

### 3.2. Biosensors Based on Carbon Nanotubes

According to the literature, CNT-based electrodes have been widely used for the development of biosensors by immobilizing enzymes and other composites on the electrode surface, for the detection of biomolecules [87,88,89]. The electrochemical response of a biosensor depends on the nature of the electrode and on the compound immobilized on it. The most commonly used enzymes used to develop biosensors for CA are tyrosinase and laccase.

**Scheme 2:**
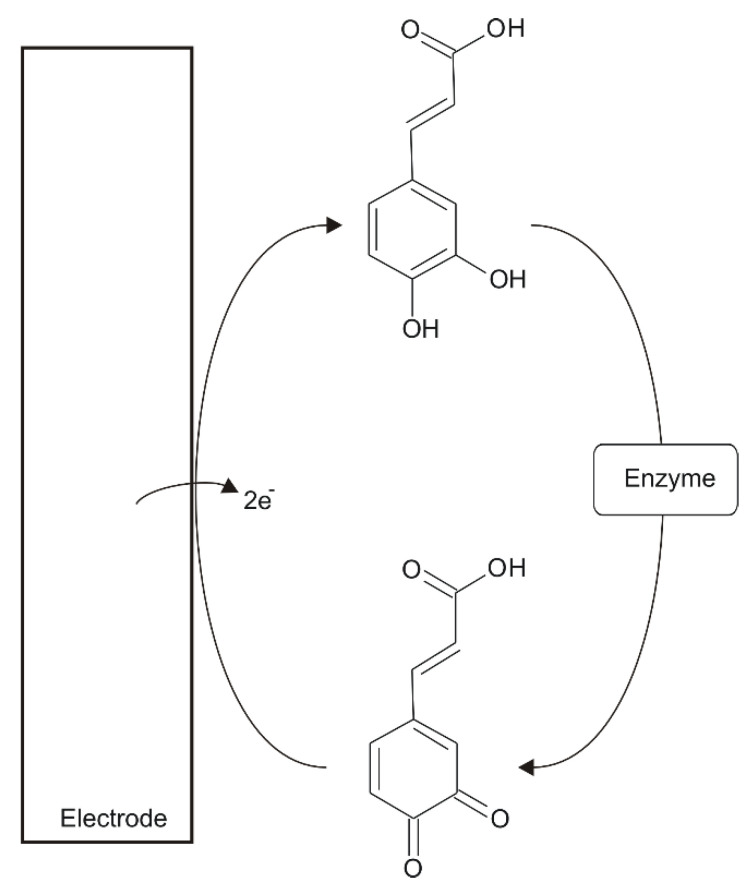
Principle of caffeic acid detection of enzyme-based biosensors.

Such biosensors were developed by Y. Vlamidis et al. in a recent study. Two amperometric biosensors were developed, based on a vitreous carbon electrode modified with reduced graphene oxide (GO) and multiwalled carbon nanotubes. Laccase (Lacc) and tyrosinase (Tyr) were also immobilized on each modified electrode in order to obtain two biosensors. Prior to enzyme deposition, GO was electrochemically reduced by cyclic voltammetry. The immobilization of enzymes on the modified electrode was optimized using various compounds (bovine serum albumin and glutaraldehyde as crosslinking agent, chitosan, and Nafion) and techniques. The best enzyme immobilization system was found to be the bovine serum albumin with glutaraldehyde for laccase and chitosan for tyrosinase. In addition, the manufacturing and storage conditions of the modified biosensors were adjusted for better stability and sensitivity. Subsequently, biosensors were used to determine catechol and other phenolic compounds, including CA. The analytical performances of the Tyr-based and the Lacc-based biosensor were compared [90]. The detection mechanism of biosensors based on Tyr or Lacc on the detection of caffeic acid is shown in Scheme 2.

Tyrosinase and laccase, which are phenol oxidases, catalyze the oxidation of caffeic acid (o-diphenol) to o-quinone derivative. This o-quinone derivative is electrochemically reduced at the electrode surface. The reduction of quinone on electrodes generate the caffeic acid that is re-oxidized by the enzyme. In this way an amplification cycle of the current is generated. The increment of sensitivity depends on the nature of the electrode and on the nature of the analyte [91].

**Figure 3:**
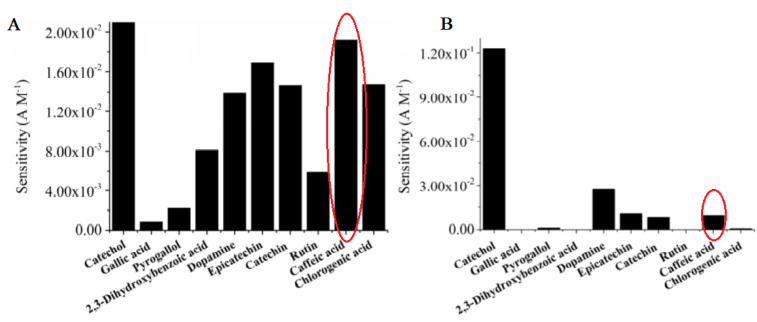
Sensitivity of Lacc-(**A**) and Tyr-(**B**) based biosensors to tested analyzes [90]. In red elipses are highlighted the results obtained in the case of the CA detection. Reprinted with the permission of the publisher.

As it may be seen in Figure 3 [90], the sensitivities obtained for the tested analytes are much lower as compared to that for catechol. Moreover, in the case of the tyrosinase biosensor, gallic acid, 2,3-dihydroxybenzoic acid, and rutin show no response. This result may be attributed to their ability to inhibit tyrosinase, possibly by blocking electron transfer, a result which is in agreement with the literature [92,93]. The poor response of the biosensor to the other analytes tested, compared to the Lacc-based biosensor, may be due to the lower activity of the immobilized Tyr.

The stability of the tyrosinase biosensor proved to be lower (one or two days) as compared to the Lacc-based biosensor (one month). This result is explained by the fact that Tyr is an unstable enzyme. Numerous studies have attempted to develop appropriate immobilization matrices to ensure the operational stability of Tyr biosensors [94]. Finally, the practical applicability of biosensors was demonstrated by estimating the concentration of total polyphenols in juice samples [90].

**Figure 4:**
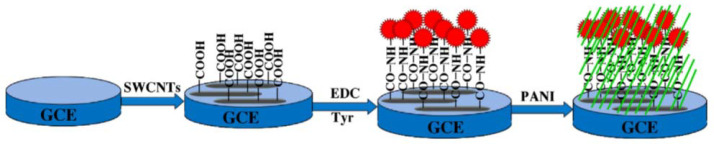
The sandwich structure of the biosensor PANI/Tyr-SWCNTs/GCE [95]. Reprinted with the permission of the publisher.

As compared to multilayer carbon nanotubes (MWCNTs), single-layer ones (SWCNTs) have superior electronic properties. They are usually arranged in a regular pattern and in contact with each other. In addition, SWCNTs may interconnect with different types of biomolecules, while they are normally aligned to the electrode surface by self-assembly and act as nanoelectrodes. SWCNTs were used to make a sensitive biosensor for the determination of phenolic compounds along with tyrosinase (Tyr) and polyaniline (PANI). The biosensor is the sandwich type, as tyrosinase is surrounded by SWCNTs and PANI [95] (Figure 4).

Surface morphologies were studied by scanning electron microscopy (SEM). The linearity range of the CA biosensor was 2.5 × 10^−7^–4.7 × 10^−4^ M and the detection limit was 6.0 × 10^−8^ M. The biosensor also showed sensitivity, good repeatability, and stability. The biosensor was applied to detect catechol or CA in real samples with satisfactory results.

The advantages of the biosensor are attributed to the components: (1) SWCNT plays an important role in immobilizing Tyr and PANI; (2) in the sandwich biosensor, PANI is introduced into the three-dimensional space of the proposed Tyr-SWCNTs/GCE, facilitating the transfer of electrons between Tyr and the electrode surface; (3) the synergistic effect of SWCNT and PANI improves the electron transfer capacity of the system and amplifies the signal [95].

### 3.3. Carbon Nanofiber Sensors

Carbon nanofibers (CNFs) are used for different electrodes, in many applications, due to the surface area/volume ratio which is higher than that of CNTs [9]. CNFs have similar conductivity and stability to CNTs. The unique chemical and physical properties make CNFs suitable materials for the development of electrochemical sensors [82].

M. Sakthivel et al. functionalized carbon nanofibers by using a cobalt-iron diselenide bimetallic nanosphere. The composite (CoFeSe_2_/f-CNF) was synthesized by a simple hydrothermal method and was used as an electrode material for efficient electrochemical detection of CA. CNF functionalization and CoFeSe_2_/f-CNF formation were successfully examined by using infrared spectroscopy, Raman spectroscopy, X-ray diffraction, transmission electron microscopy and scanning electron microscopy, and energy-dispersive X-ray spectroscopy. In addition, the electrochemical properties of the CoFeSe_2_/f-CNF (GCE) modified vitreous carbon electrode in the detection of CA by cyclic voltammetry, differential pulse voltammetry, and electrochemical impedance spectroscopy were investigated. The results of the electrochemical studies of CoFeSe_2_/f-CNF/GCE showed that the sensor has a very low detection limit (0.002 pM) and a good sensitivity (2.04 µM^−1^cm^−2^) in the CA analysis. Moreover, it was demonstrated that the CoFeSe_2_/f-CNF/GCE sensor may be used for the feasible detection of CA in red wine samples, which revealed its practical usefulness [9].

A novel study compared three screen-printed sensors based on carbon nanomaterials (carbon, carbon nanofibers, and multiwalled carbon nanotubes) to determine CA by using cyclic voltammetry. A better sensitivity was obtained by using the sensor based on carbon nanofibers. The detection limit obtained was 3.23 × 10^−9^ M. In order to demonstrate its usefulness, this sensor was successfully used for the quantitative determination of CA in three dietary supplements. The results were validated using the Folin–Ciocalteu spectrophotometric method [62].

### 3.4. Biosensors Based on Carbon Nanofibers

Carbon nanofibers have, in particular, high mobility of load carriers along the axis of rotation, they are easy to process, and have good chemical stability. However, the semiconductor properties of unmodified carbon nanofibers are not sufficient to achieve biosensors with superior analytical performance. The semiconductor properties of carbon nanofibers may be improved by chemical doping [96]. Chemically doped CNFs have been used to detect several analytes [96,97,98]. Carbon nanofiber biosensors have been reported for a number of antioxidants [99,100,101,102] but not for the CA.

### 3.5. Graphene-Based Sensors

Graphene has been used for the development of sensors and biosensors in many applications, especially due to its properties which facilitate the electrochemical determination of analytes. Graphene is less expensive as compared to other carbon materials, it has very good electronic properties, high electrocatalytic activity, good mechanical strength, and high thermal conductivity [103]. Modified graphene-based electrodes have attracted enormous interest in recent years and they have been successfully used for the study of organic and biological molecules [104], glucose [105], hydrogen peroxide [106], dopamine [107], methanol [108] nitrite [109], melatonin [110], rutin [111], and nitrobenzene [112]. The graphene-based sensors used to detect CA will be presented in this paper.

A work of interest, characterized a chemically modified vitreous carbon electrode with reduced graphene oxide (CRGO) for the electrochemical detection of CA. In order to characterize the electrode properties, CV, DPV, amperometry, and electrochemical impedance spectroscopy (EIS) techniques were used. The change showed a marked improvement in sensor response and a decrease in CA oxidation potential. Under optimal conditions, CRGO had a linearity range of 1 × 10^−8^–8 × 10^−4^ M and the detection limit was 2 × 10^−9^ M (S/N = 3), with a sensitivity of 192.21 μA·mM^−1^·cm^−2^ at an applied potential of +0.2 V (relative to Ag/AgCl), suggesting that CRGO is a promising sensor for the electrochemical determination of AC. The results showed the high sensitivity, selectivity, and reproducibility of the electrode modified with reduced graphene oxide. In addition, the modified electrode was used for AC detection in wine samples with optimal results. The modified CRGO electrode had good stability. Oxidation peaks retained 95.7% of the initial response and no obvious potential change was observed, indicating that the modified CRGO electrode has long-term stability [113].

The gel made of graphene and ionic liquid deposited on a working electrode made of carbon paste represents another possible approach to determining the CA. The gel was synthesized, electrochemically deposited, and characterized from a morphological and structural point of view. For this purpose, several analytical techniques were applied, such as HR-TEM/EDX (high resolution electron microscopy/electronic transmission/energy scatter X-ray analysis); FE-SEM/EDX (electron scanning emission-scanning field), XPS (X-ray photoelectron spectroscopy); FT-IR (Fourier transform-infrared spectroscopy), and electrochemical techniques. The sensor performance was better in the concentration range of 0.025–2.00 M, demonstrating good reproducibility, with a sensitivity value of 3389 μA·mM^−1^·cm^−2^, fast response time (2 s), and a detection limit of 0.005 mM for the detection of CA. This nanocomposite gel is a prototype of a nanostructured and miniaturized sensor, useful for the “in situ” quantification of a biologically important molecule, such as CA [114].

Along with the reduced graphene oxide, polymers have the role of improving the properties of the modified sensor. Thus, K. Thangavelu’s work characterizes an electrochemical sensor for CA detection by using a vitreous carbon electrode modified with reduced graphene oxide and a polydopamine composite (PDA). The electrode was initially modified with PDA and the graphene oxide was next reduced by using the electrochemical method, the RGO/PDA composite being obtained. The RGO/PDA composite was used as an electrocatalyst for CA oxidation. Cyclic voltammetry was used to investigate the electrochemical behavior of several electrodes modified from CA oxidation and the CV results showed that the RGO/PDA composite has superior electrocatalytic activity. Differential pulse voltammetry was used to determine the CA and showed that the response to the CA was linear in the 5.0 nM and 450.55 μM concentration range, with a low detection limit (1.2 nM). The results indicate that the modified electrode has an acceptable selectivity in the presence of interfering species. The practical applicability of the sensor was evaluated on wine samples, and the percentage of analytical recovery of CA in such samples certifying the potential of this sensor in practical applications [115].

**Figure 5:**
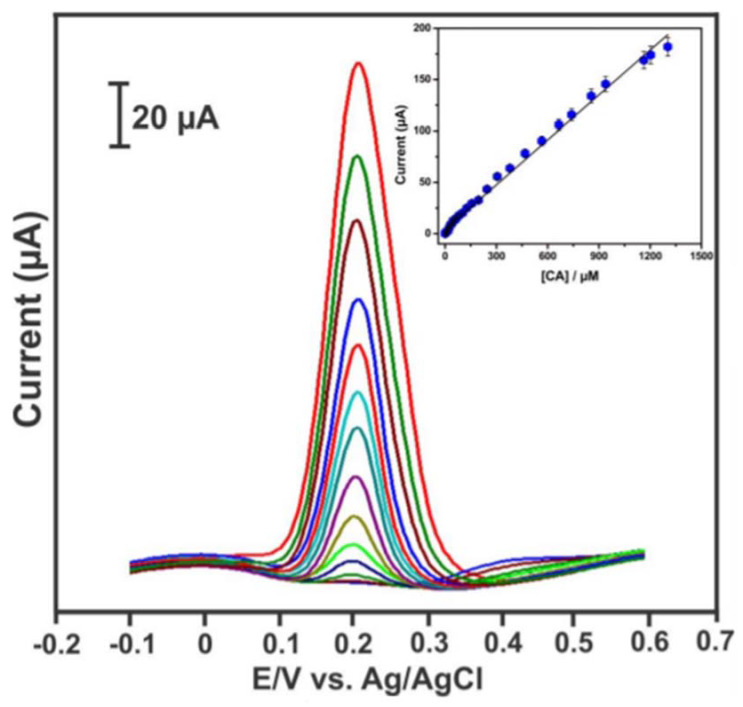
DPV response of the modified Au/PdNPs-GRF/GCE electrode for different CA concentrations from 0.03 to 938.97 μM at pH 7.0. Inset is linear plot for DPV current response vs. [CA] [70]. Reprinted with the permission of the publisher.

The use of noble metals in the form of nanoparticles could represent another means of improving the behavior of graphene. One paper dealt with the simultaneous electrochemical deposition of gold and palladium nanoparticles on graphene flakes (Au/PdNPs-4 GRF) for the sensitive determination of CA. The physicochemical properties of Au/PdNPs-GRF have been characterized by the use of numerous analytical techniques, such as scanning electron microscopy, electron scattering X-ray spectroscopy, infrared/Fourier transform spectroscopy, X-ray diffraction, Raman spectroscopy, and electrochemical impedance spectroscopy. The electrochemical techniques used for CA detection were cyclic voltammetry and differential pulse voltammetry. As a result, the modified Au/PdNPs-GRF electrode showed an excellent electrocatalytic activity as compared to CA, with a wide linearity range (0.03–938.97 pM), as it may be seen in Figure 5 and a low detection limit (6 nM) [70].

Au/PdNPs-GRF was shown to be a selective and stable material for CA detection. In addition, the proposed sensor showed adequate results in the analysis of wine samples, with very good recoveries and without significant interference [70].

Graphene doping and GO with heteroatoms (e.g., F, Cl, N, B, and S) at molecular and atomic levels favorably improves electrochemical behavior and electrocatalytic properties, as well as electronic and physicochemical properties [116,117]. Depending on the nature of the dopants and on their binding conformations, the characteristics of graphene may be improved and may have multiple advantages in various applications. Doping GO with F or its F-GO derivatives has intensified research due to their exceptional properties (e.g., high temperature resistance, improved electrocatalytic activity, and remarkable chemical inertia) [118,119]. Graphene doping with F modifies the electronic structure due to the incorporation of sp^3^ carbon into the sp^2^ honeycomb structure of the graphene [120]. This change in its electronic structure could be explained by the withdrawal of electrons and the electron donation of fluorine atoms, due to the strong electronegativity and the presence of nonparticipating electrons [121]. Consequently, F-doped graphene derivatives are useful in a number of applications, such as supercapacitors, batteries, and biomedical devices [122,123,124,125].

A recent paper described an electrochemical sensor developed by using fluorine-doped graphene oxide (F-GO) for the detection of CA. Synthesized graphene oxide (GO) and F-GO nanomaterial were characterized by scanning electron microscopy (SEM), and the presence of semi-ionic bonds was confirmed in F-GO by using X-ray spectroscopy. The electrochemical behaviors of the vitreous carbon electrode (GCE), F-GO/GCE and GO/GCE were compared by using CV, and the results obtained revealed that F-GO/GCE showed the highest electrochemical activity. DPV was used for the analytical quantification of CA, and F-GO/GCE produced a stable oxidation signal in the concentration range of 0.5–100.0 µM, with a low detection limit of 0.018 pM. Moreover, the results obtained from selectivity studies revealed a strong anti-interference capacity of F-GO/GCE in the presence of other hydroxycinnamic acids and of the ascorbic acid. F-GO/GCE provided good sensitivity, long-term stability, and excellent reproducibility. The practical applicability of the F-GO electrochemical sensor has been verified by using various brands of commercial wine, without the need for pretreating the samples. The results showed that the modified sensor may be used to control the quality of food and beverages [126].

Metal-organic compounds (MOFs) are a new class of porous polymers obtained from metal ions (or clusters) and organic molecules [127]. The large surface area of MOFs may increase the sensitivity of the sensor. Among the various MOFs, bimetallic MOFs have attracted interest in the field of sensors. It has been found that the incorporation of a second metal into the polymer may create different active catalytic sites, improving the optoelectronic, magnetic properties [128,129]. However, little research has been undertaken on the electrochemical detection of sensors based on bimetallic MOFs. MOF-818 is a new bimetallic Cu-Zr compound, which Yu Yan and colleagues used in a paper to develop an electrochemical sensor [130].

The bimetallic MOF-818 composite and the reduced graphene oxide matrix/multiwalled nanotubes (RGO/MWCNTs) were successfully synthesized by a simple solvothermal method. Characterization by electron scanning microscopy, X-ray diffraction, adsorption–desorption isotherm of N2, led to the conclusion that the composite MOF-818/RGO/MWCNTs has a porous structure, good electrical conductivity, and many active sites. MOF-818/RGO/MWCNTs/GCE was tested for the detection of phenolic compounds: CA, chlorogenic acid (CGA), and gallic acid (GA).

The sensor has two linearity ranges between 0.2 and 7 μM and 7–50 μM with a high sensitivity of 12.89 μA/μM for CA detection and a low detection limit of 5.7 nM. The sensor has also been used for determinations in serum and urine samples, proving that it has good stability and reproducibility [130].

### 3.6. Graphene-Based Biosensors

Graphene-based nanocomposites have been used successfully for high yield and more stable immobilization of oxidoreductive and hydrolytic enzymes [131,132,133]. Enzymes immobilized by both adsorption and covalent bonds on graphene nanocomposites were more stable to different types of denaturants and less sensitive to their specific inhibitors [131,134]. Enzymes immobilized on nanocomposites showed very high specificity, selectivity, wide ranges of linearity and sensitivity, and a very low detection limit [135]. The results showed that, in numerous cases, the activity of the enzymes was remarkably improved when immobilized on such supports. Graphene-based biosensors have been used to detect compounds (acetylcholine, H_2_O_2_, CA, captopril, glucose, histamine) in clinical, environmental, or food samples [136,137,138,139,140,141,142].

In 2016, I. Vasilescu et al. proposed as a support for the immobilization of laccase, a nanocomposite made up of molybdenum disulfide (MoS_2_) and graphene quantum dots (GQDs). It was observed that the conductivity of screen-printed carbon electrodes was improved after modification with nanofoils of MoS_2_ and GQDs, becoming a compatible matrix for laccase immobilization. The biosensor was used to detect CA. The influence of electrode changes on electroanalytical performance was evaluated by UV-Vis and fluorescence absorption spectroscopy, electron scanning microscopy, electron transmission microscopy, X-ray diffraction, electrochemical impedance spectroscopy, and cyclic voltammetry. The home-based biosensor developed in this study effectively detected CA in a concentration range of 0.38–100 pM, with a detection limit of 0.32 pM and a sensitivity of 17.92 nA·pM^−1^. The proposed analytical method was successfully applied to determine the total polyphenol content of red wine samples [139].

A single-use amperometric biosensor was developed to detect total polyphenolic compounds in tea infusions. The biosensor was designed by modifying the surface of a screen-printed carbon electrode with platinum nanoparticles and reduced graphene oxide, followed by immobilization and stabilization of laccase in neutral 1% Nafion solution. The obtained biosensor was characterized by electron scanning microscopy and electrochemical techniques. It was observed that the reduced graphene oxide—platinum nanoparticles composite had synergistic effects on electron transfer and increased the electroactive surface of the screen-printed electrode. The analytical method performed showed a good linearity in the range of 0.2–2 μM for CA and a detection limit of 0.09 μM. This single-use laccase biosensor could be a valuable tool for estimating the total polyphenol content of tea infusions [143].

Recently, a simple and highly sensitive electrochemical biosensor based on an immobilized laccase on a screen-printed carbon electrode modified with gold nanoparticles and graphene nanoplatelets (Lacc/AuNP/GNPl/SPCE) was developed to determine hydroquinone (HQ) and other phenolic compounds, including CA. Interference studies of most common compounds with the determination of hydroquinone have shown negligible effects.

Finally, the analytical method using the biosensor was applied to determine the antioxidant capacity in wine and affine syrup and compared with the Trolox spectrophotometric method (6-hydroxy-2,5,7,8-tetramethylchromane-2-carboxylic acid). The results were satisfactory and in agreement with the results obtained by the reference method (the Trolox equivalent antioxidant capacity—TEAC test) [144].

### 3.7. Sensors Based on Conductive Polymers and Carbon Nanomaterials

Poly (3,4-ethylenedioxythiophene) (PEDOT) is one of the conductive polymers (CP) stable in solutions, suitable for facilitating the oxidation of many molecules in drugs, food, biological fluids, etc. PEDOT has stood out due to its important properties for electrochemical analyses: high electrical conductivity, superior optical transparency, long-term electrochemical stability, and extremely high mechanical strength [145]. The PEDOT layer provides a rapid electron transfer process to the electrochemical oxidation of CA [146,147].

The synthesis of PEDOT in aqueous medium may be performed by using a water-soluble polyelectrolyte as emulsifier, for example poly (styrene-4-sulfonate) (PSS) [148].

A recent study focused on the intrinsic role of poly (3,4-ethylenedioxythiophene) (PEDOT) in the electrooxidation of CA to modify a vitreous carbon electrode. The influence of the thickness of the electrosynthetized PEDOT layers was studied, as well as the influence of the added doping compounds (sodium dodecylsulfate and sodium polystyrene sulfonate (PSS)). Depending on the scan rate, it was found that CA diffuses into the thick layers of polymer and absorbs into the thin layers. This effect could be correlated with the increase in the number of electroactive sites available as the thickness of the polymer layer increases.

Regarding the electroanalytical characteristics, it was found that, depending on the adsorption of the polymer, a higher electroanalytical sensitivity may be obtained in a more restricted linearity range. In order to control the adsorption, a hyperbolic equation of the Michaelis–Menten type is used to model the nonlinear dependence as a function of concentration. The use of a nonlinear calibration curve provides an extended range of concentrations for electroanalytical determinations under well-established adsorption conditions. Doping compounds (polystyrene sulphate and dodecyl sulphate) were found to have no effect on the electroanalytical performance of PEDOT for CA oxidation [149].

A new and sensitive method for the fluorescent determination of CA was suggested by using silane-operated quantum dots carbon, coated with molecularly printed polymers (CDs/MIPs). The biosensor was constructed by a sol–gel process. The prepared CDs/MIPs were characterized by infrared spectroscopy, electron transmission microscopy, and fluorescence spectroscopy. The CDs/MIPs showed a distinct selectivity and a high binding affinity for CA, as well as a good reproducibility. Under optimal conditions, the fluorescence intensity of CDs/ MIPs decreased linearly with increasing CA concentration in the range of 0.5–200 μM and the detection limit was 0.11 μM. Finally, the proposed method was successfully applied to determine CA in human plasma. The result indicates that the method has a high potential when monitoring the concentration of CA for medical purposes [150].

The recent trend is to combine CP (and especially PEDOT) with several components, for example metal nanoparticles (Au, Pd, Pt, etc.) and carbon materials (carbon, carbon nanotubes or reduced graphene oxide). This allows new properties to be discovered due to the synergistic effect, such as large surface area, hydrophobic interaction, good biocompatibility, and additional functional groups. Up to the present, various nanomaterials have been incorporated with PEDOT, including metal nanoparticles, redox mediators, ionic liquids, polymers, DNA [151,152,153,154]. PEDOT-based nanocomposites benefit from the properties of each component by providing improved performance to the electrochemical sensor [155].

**Figure 6:**
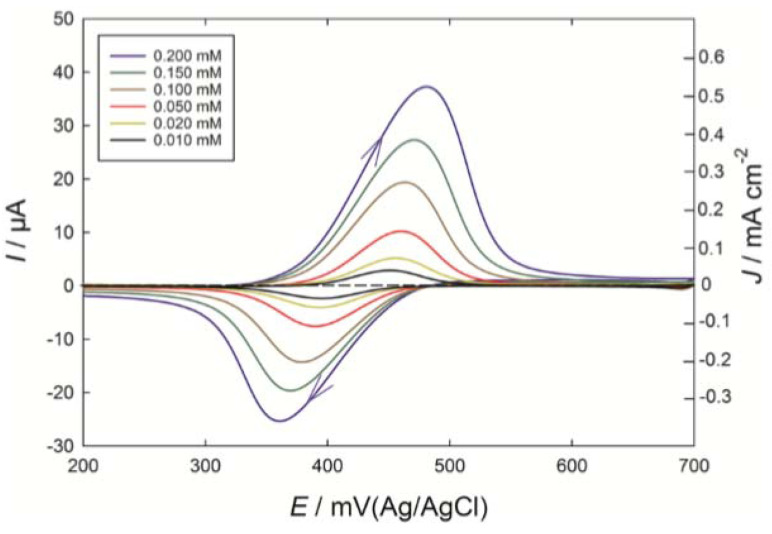
Cyclic voltammograms at a PEDOT electrode of phenolic standards in the concentration range from 0.01 to 0.20 mM, dissolved in the pH 3.6 model wine solution: (a) CA taken to 700 mV. [156].

In another research, the conductive polymer PEDOT was polymerized on microelectrodes from a solution of propylene carbonate for the detection of phenolic compounds. The electrodes were tested in a Chardonnay wine, and in model wine solution. PEDOT-based electrodes were compared to vitreous-based electrodes. A well-defined anodic peak was observed for white wine in the PEDOT analysis in the range of 400–450 mV (Ag/AgCl). The peaks obtained corresponded to the CA and catechins present in the sample to be analyzed (Figure 6).

A considerable preconcentration of phenolic compounds occurs when PEDOT-based electrodes are used. With the help of stripping voltammetry, the phenolic acids present in beverages such as wines and teas may be quantified. The very high surface obtained with the help of PEDOT indicates the possibility of developing extremely sensitive microelectrodes. Further research is needed to optimize experimental conditions, for example accumulation time, and correlations with independent measurements of phenolic composition [156].

### 3.8. Biosensors Based on Conductive Polymers and Carbon Nanomaterials

The use of electroanalytical techniques in combination with enzymes ensures adequate selectivity for the determination of polyphenols, such as CA. In this context, enzymatic biosensors have been extensively studied to determine polyphenols in beverages or foods [157,158,159]. Tyrosinase and laccase have been frequently used due to their acting on the same types of compounds, for example monophenols and *o*-diphenols, which are related to the antioxidant capacity of beverages [160,161,162]. On the other hand, biosensors have required constant improvement through various approaches. A typical improvement occurs by depositing a layer of conductive polymer on the surface of the electrode, thus increasing the electrochemical properties of the material; in addition, this layer facilitates and supports the immobilization of the enzyme [161,163]. The electrodeposition of conductive polymers (CP) for biosensors is usually performed by classical potentiostatic and/or galvanostatic methods or by superimposing a sinusoidal current over a constant direct current [161,164,165,166,167]

**Figure 7:**
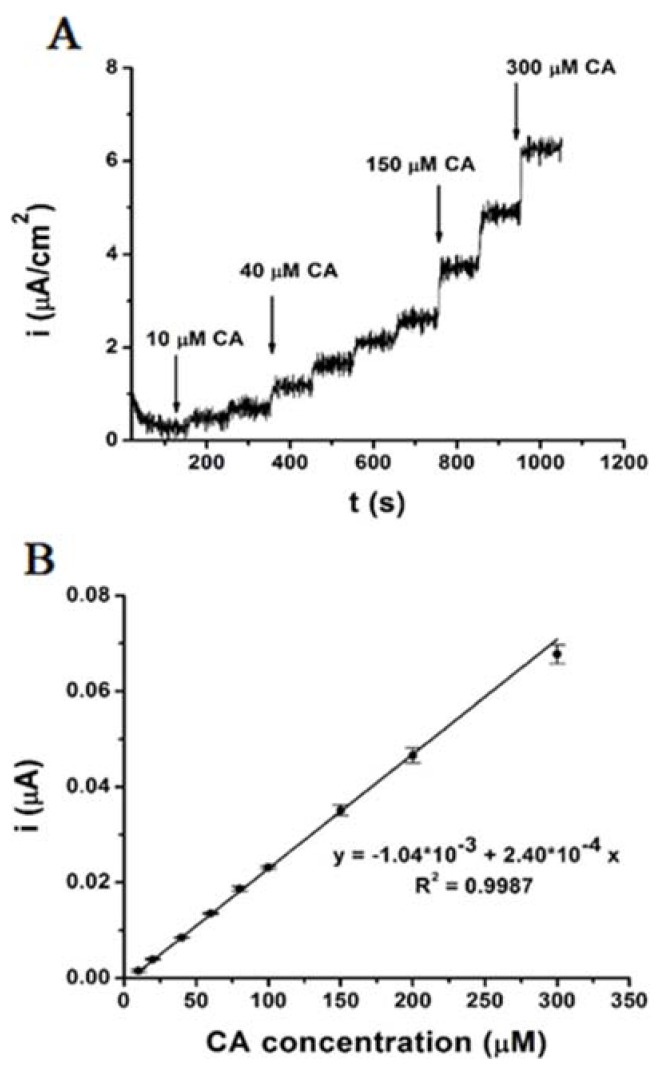
(**A**) Chronoamperogram corresponding to CA additions (10, 20, 40, 60, 80, 100, 150, 200, and 300 µM) at the PEDOT-Tyr/SNGC biosensor; (**B**) Calibration plot with error bars expressed as standard deviation.

Such a method was addressed in a recent study for the development of a tyrosinase and PEDOT-based biosensor. The poly (3,4-ethylenedioxythiophene)-tyrosinase/Sonogel-Carbon biosensor (PEDOT-Tyr/SNGC) was proposed for the analysis of beer and wine. In order to produce this biosensor, the method of electrodeposition with sinusoidal current superimposed over a constant direct current was used. This method ensures enzyme immobilization, a technique which generates a nanostructured surface. The biosensor was electrochemically characterized, by using cyclic voltammetry and electrochemical impedance spectroscopy. Chronoamperometry was used for quantitative determinations with the biosensor. Figure 7A shows the chronoamperogram obtained by adding different concentrations of CA (10–300 pM). The calibration curve was made from the chronoamperogram, by using the current increase with each addition of CA (Figure 7B). The limits of detection, quantification, and sensitivity for the PEDOT-Tyr/SNGC biosensor to CA were 4.33 pM, 14.43 pM and 2.40 × 10^−4^ µA·µM^−1^, respectively. Repeatability of the measurements was evaluated in the range of concentrations 10–300 µM, obtaining a relative value of the relative standard deviation (RSD) of 2.67% [161].

A sample of nine types of beer and four types of wine (three red wines and a white one) was tested for validation. Polyphenol concentrations for beer and wine were evaluated by using the suggested biosensor, the values obtained being in accordance with the data reported in the literature. The antioxidant properties of the samples were also evaluated by using the spectrophotometric method for capturing 2,2-azino-bis-(3-ethylbenzothiazoline-6-sulfonic acid) (ABTS) radicals. The PEDOT-Tyr/SNGC biosensor developed in this study was demonstrated to special properties in evaluating the polyphenolic composition and antioxidant capacity of the tested beverages [161].

In 2019, a group of researchers developed a new biosensor by immobilizing the laccase by a potentiostatic deposition in a thin layer of polydopamine (ePDA) on the surface of the carbon electrode. The morphology, wettability, optical, and electrochemical properties of the modified electrode were analyzed by atomic force microscopy, water contact angle measurement, ellipsometry and cyclic voltammetry. The results indicate that laccase is strongly immobilized and evenly distributed in the ePDA matrix and does not significantly affect the redox behavior of the polymer, suggesting physical interactions between the immobilized enzyme and ePDA, as well as a good enzyme distribution on the sensor surface. In contrast, the presence of the enzyme favored electron transfer and less hydrophilic behavior as in the case of plain ePDA film. The catalytic activity of polymerase-modified electrodes was confirmed by using chronoamperometry for the detection of 2,2′-azino-bis-(3-ethylbenzothiazoline-6-sulfonic acid) (ABTS), a known laccase substrate. The electrochemical method of biofunctionalization has also been used successfully to modify single-use graphite electrodes, obtaining selective and highly reproducible responses to several phenolic compounds: caffeic, rosmarinic, and gallic acid. The biosensor demonstrated its effectiveness for the detection of CA by the detection limit of 0.14 μM, in a linear range of 1–50 μM and a sensitivity of 81 μA cm^−2^ mM^−1^. This simple but efficient method of modifying electrodes has great potential in monitoring existing phenolic compounds in industrial waste [168].

### 3.9. Carbon Black Sensors

Carbon black (CB) is an amorphous carbon produced by incomplete carbon combustion, for example pyrolysis of industrial carbon. The most relevant method of industrial production involves the partial combustion of liquid aromatic hydrocarbons in a furnace, providing black particles with nanometric diameters, which form millimeter-sized aggregates [169].

CB may be used in the form of paste or ink to modify the electrodes, by the technique of casting, screen printing, or printing. Over the years, various phenolic compounds have been determined by using CB: NADH, thiols, ascorbic acid, CA, gallic acid, hydrazine [170,171,172,173,174,175]. CB is able to improve electron transfer, sensitivity to the determined analyte, resistance to contamination, and to decrease the potential applied for the quantification of analytes [176], after deposition on various electrodes (carbon screen-printed, glassy carbon, or carbon paste) [177,178,179].

The use of CB does not require additional chemical treatment (the use of strong oxidant is avoided as in the case of carbon nanotubes), is cost effective (about 1 € for 1 kg) [170] and may be dispersed in solution, having good stability. This explains the interest regarding the use of CB in the detection of CA [175].

D. Compagnone and the team used CB to modify the vitreous carbon electrodes so as to achieve improved performance in terms of stability, sensitivity, and reproducibility. The main advantage of the CB-modified GC electrode lies in its passivation resistance, which leads to the possibility of reusing the electrode for dozens of repeated determinations. The obtained electrodes were tested for the determination of monophenols (tyrosol) and *ortho*-diphenols (caffeic acid). Moreover, CB improves catalytic performance by shifting the potential to lower values. This, combined with an enlarged electrode surface, leads to higher sensitivity. The study demonstrated the feasibility of modifying the surface of the vitreous carbon electrode with CB by creating a more stable sensor, which allows the detection of phenolic compounds in olive oil samples [180].

D. Talarico and his collaborators developed a miniaturized and single-use electrochemical sensor for the detection of phenolic compounds. The sensor was constructed by modifying the surface of the screen-printed electrode (SPE) with a CB dispersion. As in the previously mentioned study, the modified sensor had a higher sensitivity and better reproducibility as compared to the unmodified SPE. The phenolic compounds catechol, gallic acid, CA, and tyrosol were determined by square wave voltammetry with a detection limit of 0.1, 1, 0.8, and 2 μM, respectively. The sensor was able to selectively determine monophenols and *ortho*-diphenols in a fast and easy way, being a cost-effective device for controlling foods and beverages containing phenolic compounds [181].

**Figure 8:**
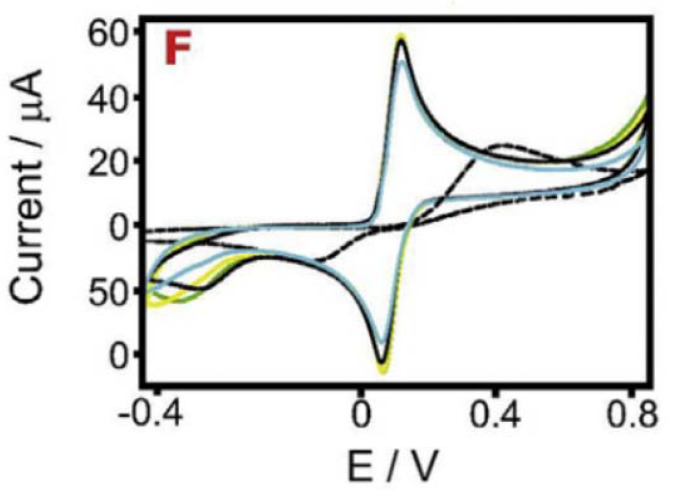
Cyclic voltammograms obtained in phosphate buffer 0.05 M-KCl-0.1 M, pH 7.4, in presence of 2 mM of CA. All recorded at a scan rate of 50 mV·s^−1^ using bare SPEs (dashed line) and SPEs modified with HP 160 (green line), N115 (yellow line), N375 (black line), and PL6 (cyan line) [169].

Speaking of miniaturized sensors, a paper from 2019 compared several types of CB used to modify the working electrode. HP160, HS20, MTN990, N115, N220, N375, N660, PL6, Super P, and XE2B are CB types which were characterized by Raman spectroscopy, transmission electron microscopy (TEM), and scanning electron microscopy (SEM). Subsequently, for the electrochemical characterization of screen-printed electrodes modified with these types of CBs, cyclic voltammetry and electrochemical impedance spectroscopy in ferrocyanide/ferricyanide solution were used, highlighting the advantage of using CB as compared to the unmodified electrode. The best results were obtained when using screen-printed electrodes modified with CB N115, N375, HP160, and PL6. They were successively tested for the detection of several analytes: epinephrine, benzoquinone, ascorbic acid, cysteine, catechol, and CA, by using cyclic voltammetry, a remarkable improvement in electrochemical performance being observed as compared to the unmodified electrode. In the case of epinephrine and CA, a separation of peaks was noticed when combined with higher intensity anodic and cathodic peaks for SPEs modified with HP160 and N115. Figure 8 shows the cyclic voltammograms obtained in 0.05 M 0.1 M phosphate buffer, pH 7.4, in 2 mM CA solution. All CVs are recorded at a scan rate of 50 mV·s^−1^ by using unmodified SPEs and modified SPEs [169].

The results showed that several types of CB may remarkably improve the performance of electrochemical sensors in terms of decreasing the applied potential or separating the peaks, of increasing the current and decreasing the resistance to electron transfer due to several key features among which nanometric dimensions, the structure of the carbonaceous material, and the large number of surface defects [169].

Recent research has focused on the manufacture of a complex sensor for the simultaneous determination of hydroxycinnamic acids: caffeic (CA), sinapic (SP), and p-coumaric acids (CM). The sensor was constructed of a screen-printed electrode modified with a nanocomposite which integrates wolfram disulfide (WS_2_) decorated with gold nanoparticles with catechin (AuNP-CT) and carbon black (CB). WS_2_ decorated with AuNP-CT in a high conductivity network of CB has excellent properties which prevent fouling, and result in high selectivity, sensitivity, and reproducibility. Using DPV, three clear oxidation peaks were observed for the target hCNs for the SPS-CB-WS_2_/AuNP-CT sensor. The sensor proved an excellent capacity in preventing fouling (RSD i_p_ ≤ 3%, *n* = 15) and low detection limits for all hydroxycinnamic acids (CA 0.10 μmol·L^–1^). The SPE-CB-WS_2_/AuNP-CT sensor was successfully applied to determine CA, SP, and CM in food samples with good accuracy (RSD ≤ 4%, *n* = 3) and good analytical recovery (86–109%; RSD ≤ 5%, *n* = 3). The proposed sensor is the first device which exploits the simultaneous determination of these compounds in food samples. Due to its excellent electrochemical performance, low cost, availability, and ease of use, this sensor modified with the nanocomposite SPE-CB-WS_2_/AuNP-CT is a useful tool for determining bioactive compounds [175].

### 3.10. Carbon Black Biosensors

It is known that biosensors are basically chemical sensors which use the recognition properties of biomolecules on the electrode surface and have been widely used to determine biological molecules, pathogens, or tumor markers, much needed in the medical field [102].

Often, carbon black (CB) nanomaterials have been used to increase electrochemical biosensitivity, rapid electron transfer, high thermal and electrical conductivity, the possibility of combining/modifying with various other nanomaterials or mediators, and reduced costs [182]. The literature describes carbon black-based biosensors for the detection of biomolecules such as glucose, DNA, proteins, catechol, bisphenol, hydroquinone, pesticides, etc. [183,184,185,186,187,188,189].

As far as we know, there are few studies on the detection of CA using a biosensor based on carbon black.

In 2013, S. Nadifiyine et al. developed a biosensor constructed by immobilizing tyrosinase on the carbon black paste electrode. This device had a low background noise as compared to enzyme sensors based on graphite paste electrodes. Biosensors with peroxidase and laccase were also prepared. The responses of these three enzymatic biosensors for 20 different phenolic compounds, including CA, were compared. The content of phenolic compounds in olive oil was assessed by reference to the activity of the 10 μM CA solution before and after the injection of 250 mL of olive oil extract [91]. The sensitivity of the tyrosinase, laccase, and peroxidase biosensors for CA was calculated by obtaining 0.22, 0.18, and 0.05 A·M^−1^·cm^−2^, respectively. A good statistical correlation was observed when comparing the results obtained by using the biosensor on tyrosinase base and the Folin–Ciocalteu spectrophotometric method for the determination of phenolic compounds in olive oil [91].

As it may be seen, there is a wide variety of electrochemical sensors and biosensors used to detect CA. In order to have a clearer picture on the research in this field and the analytical performances of (bio) sensors, the electrochemical sensors and biosensors which determine the caffeic acid made mostly in the last 10 years are presented in Table 1.

## 4. Conclusions

This paper presents recent research on voltammetric electrochemical sensors and biosensors for the detection of caffeic acid, a polyphenolic compound with remarkable antioxidant properties, which is important in food and pharmaceuticals, but also in medical analysis. The paper also describes the main voltammetric techniques used in the analyses made to determine this analyte of interest. Voltammetric electrochemical sensors and biosensors are efficient, reliable, and viable portable instruments for the rapid, accurate, and low-cost determination of caffeic acid. The most used materials for building voltammetric electrochemical sensors and biosensors are carbon nanomaterials, such as graphene, carbon nanotubes, carbon nanofibers, carbon black, etc., with important analytical properties for sensitive and selective determinations. In order to achieve outstanding performance, these materials are usually improved, modified, or doped with other compounds or nanoparticles, through various techniques such as casting, drop-and-dry, sol–gel, sonication, etc. In addition to chemical sensors, biosensors incorporate enzymes for fast, accurate, and selective detection of analytes. Most biosensors used to detect caffeic acid are based on two of the important enzymes, tyrosinase and laccase. In this paper we focused on the techniques of immobilization of enzymes on electrodes based on carbon nanomaterials, the characterization of the sensitive material, and the analytical results obtained. In addition, the stability, reproducibility, and selectivity of biosensors were pursued, which constitute challenges for their use in practice. Superior performance is usually obtained by combining enzymes with metallic nanoparticles, conductive polymers, or other chemical mediators, which are deposited on the carbon substrate.

For the validation of sensors and biosensors, most works aimed at detecting caffeic acid in real samples, more precisely in various types of drinks (beer, wine, juices, teas) or biological fluids (human plasma, urine), generally with very good results.

Future research directions for caffeic acid detection may focus on the development of sensors and biosensors supported by carbon nanofibers or carbon black, efficient mediators of electron transfer and immobilized enzymes with the simultaneous preservation of biocatalytic properties in order to increase stability, sensitivity, and selectivity. Further research is needed on the development of precise electroanalytical methods for the quantitative determination of caffeic acid in real samples and the validation of these methods so that they may become routine methods for the analysis of dietary supplements in the pharmaceutical market.

## Figures and Tables

**Table 1:** Electrochemical sensors and biosensors used for CA detection.

No.	Modifying Material	Detection Technique	Linear Range (μM)	LOD (μM)	Sensitivity (μA·μM^−1^·cm^−2^)	Reference
Sensors						
Carbon nanotubes						
1	Carbon paste modified with Bi decorated multiwalled carbon nanotubes and cetrimonium bromide (CTAB)	DPV	0.06–500	1.57 × 10^−4^	-	[8]
2	N-doped carbon quantum dots/hexagonal porous copper oxide decorated multiwall carbon nanotubes N-CQD/HP-Cu_2_O/MWCNT/GCE	DPV	0.05–43	0.004	31.85	[83]
3	Ce-TiO_2_/carbon nanotube composite Ce-TiO_2_/CNTs	CV DPV	10^−3^–10.0	3.3 × 10^−4^	I = 5.688 c + 2.744 (R2 = 0.997)	[84]
4	Titanium dioxide nanoparticles, carbon nanotubes, and cadmium telluride quantum dots TiO_2_/CNTs/CdTeQDs	LSV EIS Amp	0.5–360	0.15	(μA) = 0.20 + 0.01 [CA]/μM	[85]
5	Three-dimensional graphene-multiwalled carbon nanotube nanocomposite 3DG/MWCNTsNc	CV Amp	0.2–174	1.78 × 10^−2^	5.8308	[86]
6	Carbon/iron-based active catalyst f-MWCNTs/a-NaFeO_2_	DPV	0.1–17.2	0.002	44.6859	[69]
7	carbon screen-printed electrodes modified with multiwalled carbon nanotubes, functionalized larger diameter (FLD-MWCNT/SPEs)	CV	2.0–50	0.20	0.90351	[190]
Carbon nanofibers						
8	Bimetallic CoFeSe_2_ nanosphere in functionalized carbon nanofibers CoFeSe_2_/f-CNF	CV DPV EIS	0.01–263.96	0.002	2.04	[9]
9	Carbon nanofibers CNF	CV	0.1–40	3.23 × 10^−3^	-	[62]
10	Carbon fibers microelectrode CF	DPV	-	-	-	[191]
Graphene						
11	Reduced graphene oxide rGo	CV DPV EIS	1 × 10^−2^–8 × 10^−1^	2 × 10^−3^	0.192	[113]
12	Graphene and ionic liquids	CV	2.5 × 10^3^–2 × 10^6^	5	3.389	[114]
13	Reduced graphene oxide and polydopamine composite RGO/PDA	CV	5 × 10^−3^–450.55	1.2 × 10^−3^	2.15	[115]
14	Gold/palladium nanoparticles decorated graphene flakes Au/PdNPsGRF	DPV	0.03–938.97	6 × 10^−3^	-	[70]
15	Fluorine-doped graphene oxide F-GO	CV	0.5–10.0; 15.0–100.0	0.018	I_pa_ = 3.935·c (µM) + 1.919 I_pa_ = 0.261·c (µM) + 38.658	[126]
16	MOF-818 metal-organic framework-reduced graphene oxide/multiwalled carbon nanotubes composite MOF-818/RGO/MWCNTs/GCE	CV DPV	0.2–7 7–50	5.2 × 10^−3^	12.89	[130]
17	ZrO_2_/Co_3_O_4_/reduced graphene oxide (rGO)	DPV	2.48 × 10^−3^–0.524	0.62 × 10^−3^	-	[192]
18	Gold nanoparticles/graphene nanosheets modified glassy carbon electrode	CV	0.5 - 50	5 × 10^−2^	-	[193]
Polymers and carbonaceous nanomaterials						
19	Poly(3,4-ethylenedioxythiophene)glassy carbon PEDOT, thin PEDOT, thick	DPV	0.15–4 1.5–47.5	0.11 1.5	23.2 3.0	[149]
20	Carbon dots coated with molecularly imprinted polymers CDs/MIPs	Fluorometric assay	0.5–200	0.11	-	[150]
21	PEDOT	CV	10–200	-	-	[156]
22	Glassy carbon electrode modified with poly(glutamic acid)	SWV	4–30	1.25	i_p_ (μA) = 2.11705 + 1.0795·c (μM)	[194]
23	Nafion graphene oxide-modified glassy carbon electrode	SW-AdSV	0.1–1.5 1.5–10	9.1 × 10^−2^	-	[195]
24	Glassy carbon electrode modified with a dispersion of multiwalled carbon nanotubes (CNT) in polyethylenimine	CE-AD	9.2–140	2.8	-	[196]
Carbon black						
25	Carbon black CB	CV	-	-	-	[180]
26	Carbon black CB	SWV	1–50	0.8	20.7	[181]
27	WS_2_/catechin-capped AuNPs/carbon black-based nanocomposite SPE-CB-WS_2_/AuNP-CT	DPV	0.3–200.0	0.10	y = 0.0869x − 0.0473	[175]
28	Screen-printed electrode modified with carbon black and chitosan	CV	-	-	-	[197]
Biosensors						
Carbon nanotubes						
1	Reduced GO and MWCNTs Lac/ERGO-MWCNTs	CV	-	-	-	[90]
2	Tyrosinase, single-wall carbon nanotubes and polyaniline PANI/Tyr-SWCNTs	CV	2.5 × 10^−1^–470	6.0 × 10^−2^	0.492	[95]
Graphene						
3	Molybdenum disulphide and graphene quantum dots and tyrosinase CSPE-MoS_2_-GQDs-TvL	CV	0.38–10 10–100	0.32	17.92 × 10^−3^	[139]
4	Platinum nanoparticles-reduced graphene oxide-laccase	CV	0.2–2	0.09	2.147	[143]
5	Laccase immobilized on screen-printed carbon electrode modified with graphene nanoplatelets and gold nanoparticles Lacc/AuNP/GNPl/SPCE	CV	-	-	-	[144]
Polymers and carbonaceous nanomaterials						
6	Poly(3,4-ethylenedioxythiophene)-tyrosinase PEDOT-Tyr	CV	10-300	4.33	2.40 × 10^−4^	[161]
7	Laccase-polydopamine PDA-Lac	CV	1–50	0.14	81 ± 2 × 10^3^	[168]
8	Laccase–MWCNT–chitosan Lacc–CS–MWCNT	CV	7.35 × 10^−1^–10.5	1.51 × 10^−1^	1.277	[198]
9	Laccase–PAP–MWCNT (TvL) Laccase–PAP–MWCNT (ThL)	Amp	1.66 × 10^−7^–4.38 × 10^−6^ 1.66 × 10^−7^–3.33 × 10^−6^	5.55 × 10^−8^ 5.55 × 10^−8^	3.95 × 10^−6^ 4.21 × 10^−5^	[199]
10	Laccase–tyrosinase based sonogel–carbon	CV	1.0 × 10^−2^–2.0	2.6 × 10^−2^	167.53 × 10^−3^	[200]
Glassy carbon						
11	Laccase immobilized onto a glassy carbon electrode	Amp	5.55 × 10^−1^–5.55 × 10^−8^	3.9 × 10^−10^	-	[201]
12	tyrosinase immobilized onto a glassy carbon electrode modified with electrodeposited gold nanoparticles (Tyr-nAu-GCE)	CV	0.02–2.0 × 10^10^	6.6 × 10^13^	0.014	[202]
Carbon black						
13	Enzyme/carbon black paste electrode CBPE-Tyr CBPE-Lacc CBPE-Per	LSV Amp	-	-	0.22 0.18 0.05	[91]
